# **Mechanical properties of body-centered tetragonal lattice structures in 316L stainless steel fabricated by SLM**

**DOI:** 10.1038/s41598-026-44572-8

**Published:** 2026-03-24

**Authors:** Zhimin Xu, Zhirong Lin, Zhenzeng Wu, Huicai Xu

**Affiliations:** 1 Department of Intelligent Manufacturing Engineering, Meizhouwan Vocational Technology College, Putian, 351100 Fujian China; 2 College of Resources Engineering, Wuyi University, Nanping, 353000 Fujian China; 3 Faculty of Computer Science and Information Technology, University Putra Malaysia, 43900 Serdang, Selangor Malaysia

**Keywords:** Selective laser melting, BCT lattice structures, 316L stainless steel, Modulus of elasticity, Yield strength, Engineering, Materials science

## Abstract

Response surface methodology (RSM) was employed to investigate the mechanical performance of body-centered tetragonal (BCT) lattice structures fabricated from 316L stainless steel. A combined approach of numerical simulation and experimental validation was undertaken to explore the influence of structural parameters on mechanical properties. During the simulation stage, lattice models with different structural parameters were constructed using SolidWorks and subjected to uniaxial compression simulations in ABAQUS to obtain mechanical properties, including yield strength (YS) and modulus of elasticity (MOE). The simulation results were incorporated into the response surface model to quantitatively evaluate the influence of structural parameters on mechanical performance. To validate the numerical predictions, BCT lattice specimens with various structural parameters were fabricated using selective laser melting (SLM) and tested under uniaxial compression. The experimental results showed good agreement with the simulation results. The results indicate that both YS and MOE increase with increasing rod diameter and inclination angle, but decrease with increasing rod length. The maximum variations in YS and MOE were 143.69 MPa and 3379.54 MPa, respectively. When the rod length, rod diameter, and inclination angle were 4 mm, 1.5 mm, and 60°, respectively, the maximum YS and MOE values of 144.85 MPa and 3406.26 MPa were obtained. These findings provide a theoretical basis for the optimized design of BCT lattice structures.

## Introduction

Selective laser melting (SLM) is a representative additive manufacturing technology that has attracted increasing attention in recent years due to its ability to fabricate complex metallic components^1^. In this process, metal powders are selectively melted by a high-energy laser beam and solidified layer by layer to form three-dimensional structures. The effectiveness of SLM relies on precise control of laser energy density, which typically exceeds 10⁶ W cm⁻², ensuring complete melting of metal powders and the formation of dense structures with favorable mechanical properties^2^. The primary advantages of this technology include high design freedom and superior material utilization, enabling the realization of complex geometries and significantly reducing product development cycles^3,4^. With continuous technological advancements and gradual cost reductions, SLM technology has demonstrated immense application potential in industries such as aerospace, automotive, Energy equipment, and medical sectors^5–8^.

Lattice structures have become a key design strategy in SLM-based lightweight structures and have been widely investigated. By assembling periodic unit cells, lattice structures can effectively reduce structural weight while maintaining or even improving load-bearing capacity^9^ and energy absorption performance^10^. As a result, lattice structures are particularly suitable for applications such as support components, robotic arms, and impact-resistant structures. Common lattice configurations include cubic, tetrahedral, body-centered cubic (BCC), and face-centered cubic (FCC) structures, each exhibiting distinct mechanical characteristics under different loading conditions. The geometric freedom provided by SLM enables the practical fabrication of these complex lattice architectures, thereby promoting the development of lightweight and high-performance structural components^11^.

Among various lattice topologies, BCT lattice structures exhibit favorable structural uniformity and self-supporting capability, enabling stable fabrication without the need for additional support structures during the SLM process. The mechanical performance of BCT lattice structures is strongly dependent on their geometric parameters, including rod length, rod diameter, and the inclination angle between the rods and the reference plane. Reasonable design of these parameters can effectively tailor the stiffness, strength, and deformation behavior of the lattice structure. Therefore, understanding the quantitative relationship between geometric parameters and mechanical properties is essential for the rational design and optimization of BCT lattice structures.

Previous studies have demonstrated the advantages of lattice structures fabricated by SLM, including BCT-type architectures. Shen et al. investigated the mechanical properties and deformation behavior of ARCH and BCT lattice structures fabricated by SLM and highlighted the limitations of conventional manufacturing methods in producing such complex geometries^12^. Bai et al. reported that BCT lattice structures can maintain lightweight characteristics while exhibiting favorable mechanical performance, and established preliminary correlations between unit cell geometry and mechanical properties^13^. Wu et al. proposed optimized design strategies for variable-density lattice structures and analyzed the influence of SLM process parameters on their mechanical behavior^14^. In addition, Zhang et al. reviewed recent progress in lattice structure design and reported that advanced architectures, such as triply periodic minimal surface (TPMS) structures, can effectively reduce stress concentration and improve mechanical performance when fabricated by SLM^15^.

Despite these valuable contributions, several limitations remain in the current literature. Most existing studies focus on qualitative comparisons among different lattice topologies or investigate the influence of individual geometric or process parameters, while systematic and quantitative analyses considering the coupled effects of multiple geometric parameters are still limited. In particular, for BCT lattice structures, comprehensive investigations that integrate numerical simulation and experimental validation to establish predictive structure–property relationships are scarce. Moreover, design-oriented statistical modeling approaches that can efficiently describe parameter interactions and guide structural optimization have not been sufficiently explored for SLM-fabricated BCT lattice structures.

It should be noted that lattice structures fabricated by selective laser melting inevitably contain manufacturing-induced defects, such as surface roughness, geometric deviations of struts, and internal porosity. These imperfections may influence the mechanical response of lattice structures by inducing local stress concentration and reducing the effective load-bearing capacity. Previous studies have reported that such defects can contribute to discrepancies between numerical predictions and experimental results for additively manufactured lattice structures. Therefore, the influence of manufacturing defects should be considered when interpreting experimental results and validating numerical models.

RSM as a statistical technique based on design of experiments, provides an efficient approach to construct quantitative models describing the relationships between multiple input variables and output responses with a limited number of experiments. By incorporating interaction effects among variables, RSM enables the identification of dominant parameters and optimization pathways, thereby reducing experimental cost and improving prediction accuracy. However, the application of RSM to the mechanical performance analysis and optimization of SLM-fabricated BCT lattice structures remains limited.

To address these gaps, the present study systematically investigates the mechanical performance of BCT lattice structures fabricated from 316L stainless steel using SLM by integrating numerical simulation, experimental validation, and response surface methodology. The effects of three key geometric parameters—rod length, rod diameter, and rod inclination angle—on yield strength and modulus of elasticity are quantitatively analyzed, and their interaction effects are explicitly evaluated. By establishing statistically significant response surface models and validating them through uniaxial compression experiments, this work aims to provide a predictive framework for the design and optimization of BCT lattice structures, offering theoretical support and practical guidance for lightweight and high-performance engineering applications.

## Material and method

### Material

316L Stainless Steel power was bought from Beijing Zhonghang New Material Technology Co., LTD (Beijing, China). Phenolic plastic powder was used to optimize the performance of the model printed by 316L (Jinan Yuncheng Instrument Co., LTD, Jinan, China). Nano-TiO_2_ (Shanghai Macklin Biochemical Co., Ltd, Shanghai, China) was used to improve the performance or process effect of printing materials.

### Method

#### Establishment of BCT lattice structures

**Fig. 1:**
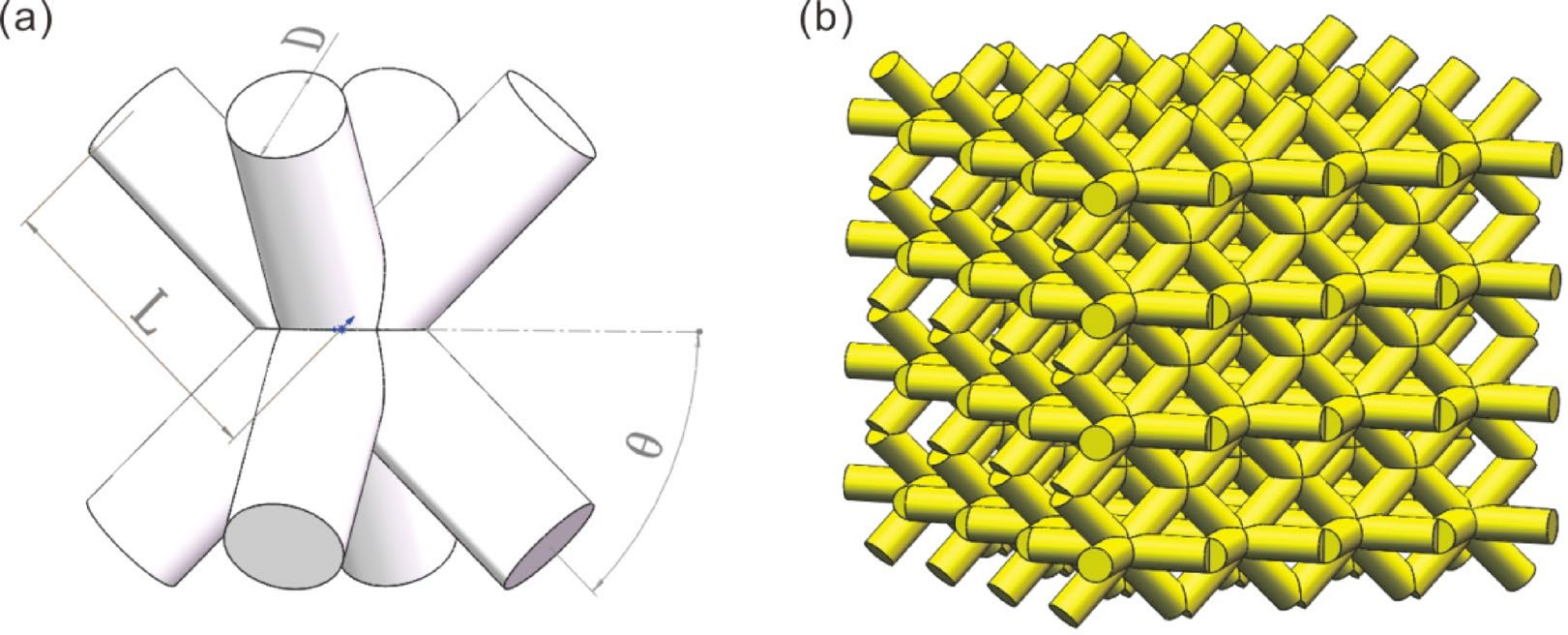
BCT structure: (**a**) unit cell and (**b**) lattice structure model.

**Fig. 2:**
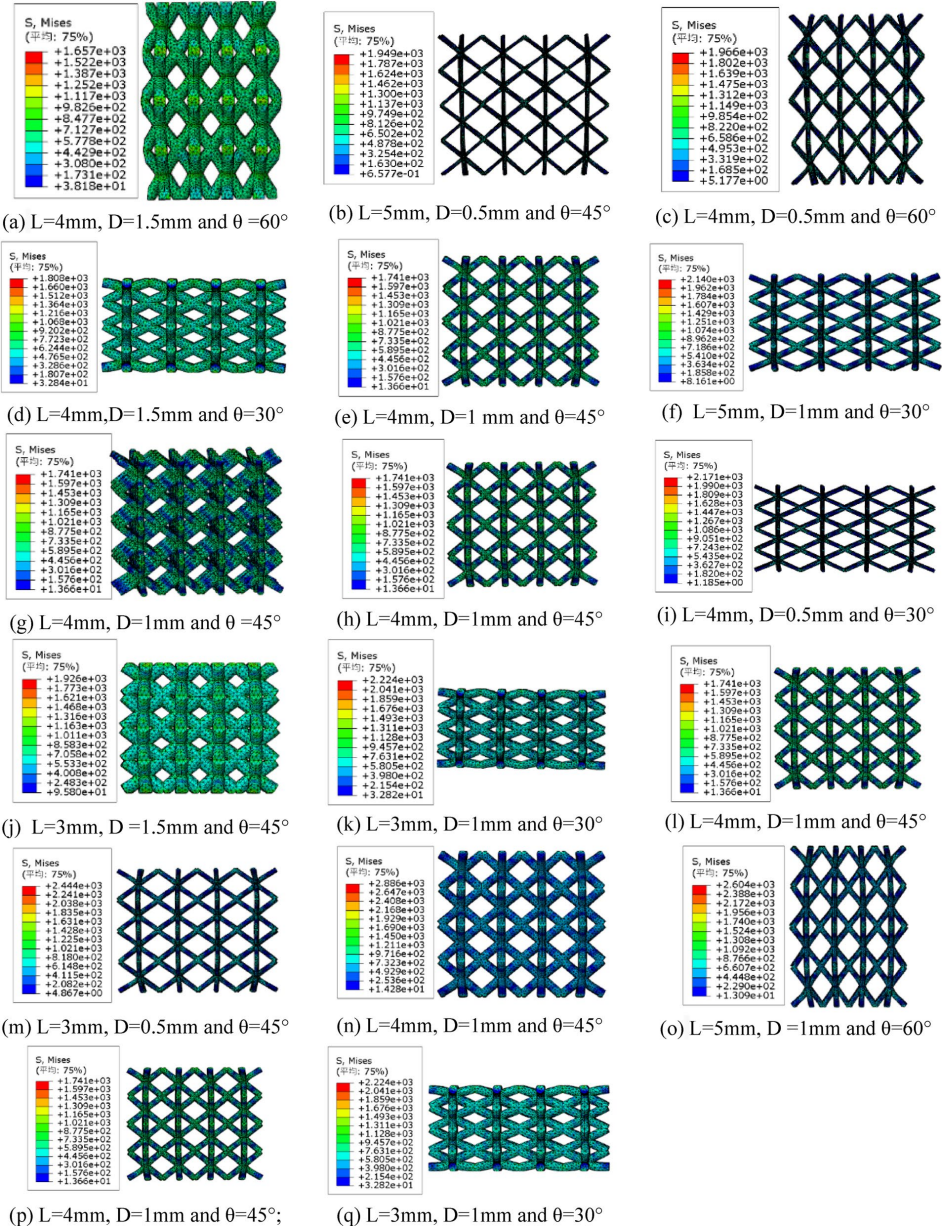
Simulation cloud maps for different structural parameters.

To thoroughly investigate the characteristics of BCT lattice structures, SolidWorks software was used to initially create three-dimensional models of single unit cells, as shown in Fig. 1. In this model, the internal structure of the BCT unit cell comprises eight rods of identical length and cross-sectional dimensions. These rods connect the body center point of the unit cell to its eight vertices, forming a robust and balanced geometric structure^16^. Arranging these single unit cells in a periodic pattern ultimately forms a complete BCT lattice structure, as illustrated in Fig. 2: BCT Lattice Structure Configuration. This 4 × 4 × 4 model demonstrates the BCT lattice structure.

#### RSM experimental design

RSM based on the Box–Behnken design (BBD) was employed to systematically investigate the effects of structural parameters on the mechanical performance of BCT lattice structures. Compared with full factorial designs, the Box–Behnken design can efficiently evaluate the main effects, interaction effects, and quadratic effects of multiple variables with a reduced number of experimental runs, thereby significantly decreasing experimental cost and time.

In this study, three key geometric parameters of the BCT lattice unit cell were selected as independent variables: rod length (L), rod diameter (D), and rod inclination angle(θ). Each factor was investigated at three levels, coded as − 1, 0, and + 1, corresponding to the low, center, and high values, respectively. According to the standard Box–Behnken design for three factors, a total of 17 trials were required.

For each experimental condition, at least two BCT lattice specimens were fabricated and tested under identical conditions, and the reported mechanical properties were obtained by averaging the measured results. In addition, five replicated tests were conducted at the center point of the Box–Behnken design to further evaluate experimental variability and ensure the reliability of the regression models. Based on the experimental data, second-order polynomial regression models were established to describe the relationships between the structural parameters and the mechanical responses, including yield strength (YS) and modulus of elasticity (MOE). The experimental design scheme is presented in Table 1.

**Table 1:** Experimental factors and levels.

Level	D (mm)	L (mm)	θ(°)
−1	0.5	3.0	30
0	1.0	4.0	45
1	1.5	5.0	60

## Results and discussion

### Simulation analysis of mechanical performance of BCT lattice structures

The simulation results are shown in Fig. 2; Table 2. Specifically, it illustrates the stress distribution under various combinations of rod length (L), rod diameter (D), and rod angle (θ). For instance, in Fig. 2a, when the rod length is 4 mm, the rod diameter is 1.5 mm, and the angle is 60°, the stress distribution within the lattice structure is uniform, indicating a high load-bearing capacity. In contrast, in Fig. 2b, when the rod length is 5 mm, the rod diameter is 0.5 mm, and the angle is 45°, the stress distribution is uneven, with localized areas of high stress concentration, suggesting weaker load-bearing capacity under these parameters.

Detailed simulation results of yield strength (YS) and modulus of elasticity (MOE) for different combinations of structural parameters is provided in Table 2. A comparative analysis reveals that both YS and MOE increase with the increase in rod diameter and angle but decrease with the increase in rod length. For example, when the rod length is 4 mm, the rod diameter is 1.5 mm, and the angle is 60°, the yield strength reaches a maximum value of 144.85 MPa, and the elastic modulus is 3406.26 MPa. Conversely, when the rod length is 5 mm, the rod diameter is 0.5 mm, and the angle is 45°, the yield strength is only 1.16 MPa, and the elastic modulus is 26.72 MPa, indicating a significant difference in performance.

**Table 2:** Response surface experimental scheme and simulation results.

No.	D (mm)	L (mm)	θ(°)	YS (MPa)	MOE (MPa)
1	1.5	4.0	60	144.85	3406.26
2	0.5	5.0	45	1.16	26.72
3	0.5	4.0	60	8.46	243.73
4	1.5	4.0	30	22.29	936.86
5	1.0	4.0	45	12.26	514.92
6	1.0	5.0	30	3.25	91.15
7	1.0	4.0	45	12.26	514.92
8	1.0	4.0	45	12.26	514.92
9	0.5	4.0	30	1.16	32.56
10	1.5	3.0	45	82.21	2549.77
11	1.0	3.0	30	18.67	798.39
12	1.0	4.0	45	12.26	514.92
13	0.5	3.0	45	4.84	159.76
14	1.5	5.0	45	18.34	701.28
15	1.0	5.0	60	20.36	965.63
16	1.0	4.0	45	12.26	514.92
17	1.0	3.0	30	18.67	701.28

The impact of different structural parameters on the stress distribution of 316L stainless steel BCT lattice structures through simulation cloud maps was visually demonstrated, revealing the comprehensive influence of rod length, rod diameter, and angle on the load-bearing capacity of the structure. Table 2 quantitatively analyzes the effects of each parameter on yield strength and elastic modulus, further validating the observations from Fig. 2. Together, they indicate that optimizing rod diameter and angle can significantly enhance the mechanical properties of the lattice structure, while increasing rod length leads to a decrease in performance. The observed dependence of mechanical properties on geometric parameters can be attributed to changes in load-transfer paths and deformation modes of the lattice struts. Increasing rod diameter enhances the effective load-bearing cross-sectional area, thereby reducing local stress concentration and improving both stiffness and strength. A larger rod inclination angle increases the axial force component along the struts under compressive loading, leading to improved resistance to deformation. In contrast, increasing rod length results in higher slenderness ratios of the struts, which promotes bending-dominated deformation and reduces the overall mechanical performance of the lattice structure.

### Simulation validation of mechanical performance of BCT lattice structures

The core objective of this experiment is to conduct an in-depth study of the mechanical performance of 316L stainless steel BCT lattice structures manufactured using Selective Laser Melting (SLM) technology, coupled with simulation analysis. Therefore, 316L stainless steel powder was selected as the experimental material. 316L stainless steel, an austenitic alloy, is known for its excellent corrosion resistance and formability. Its resistance to carbide precipitation during welding processes allows it to maintain high toughness and strength post-welding. Due to its outstanding processing performance and excellent welding characteristics, this material has been widely used in the mechanical manufacturing industry.

The forming equipment used in the experiment was the SLM-125HL metal 3D printer produced by SLM Solutions, Germany. This equipment integrates scanning, forming, control systems, and gas protection functions, with its core process being laser melting technology. During the selective laser melting of metal powders, the equipment uses traditional nitrogen as a protective gas to effectively prevent oxidation of the metal powder due to high-temperature melting. The process parameters set for the experiment are: laser power of 200 W, powder layer thickness of 0.03 mm, and scanning speed of 800 mm/s.

Compression mechanical performance tests were conducted using an INSTRON 2382 electronic universal testing machine on the 316L stainless steel BCT lattice structures. During the test, the compression speed was set to 3 mm min^−1^ to ensure smooth testing and obtain sufficient data points for subsequent analysis. The sampling frequency was set to 5 Hz to ensure the accuracy of force-displacement data. After completing the compression tests, the force-displacement curves were converted into stress-strain curves. The stress-strain curves not only clearly display the elastic stage of the material but also identify the yield point and the subsequent plastic deformation stage. Through in-depth analysis of the curves, the YS and MOE of the BCT lattice structures can be accurately determined, providing data support for further optimization of the structural design (Table 3).

**Table 3:** Chemical composition of 316L stainless steel powder.

Element	Fe	C	Si	Mn	S	*P*	Cr	Ni	Mo
Content (%)	Bal.	< 0.03	< 1.00	< 5.00	< 0.03	< 0.045	16.0–18.0	10.0–25.0	< 0.03

**Fig. 3:**
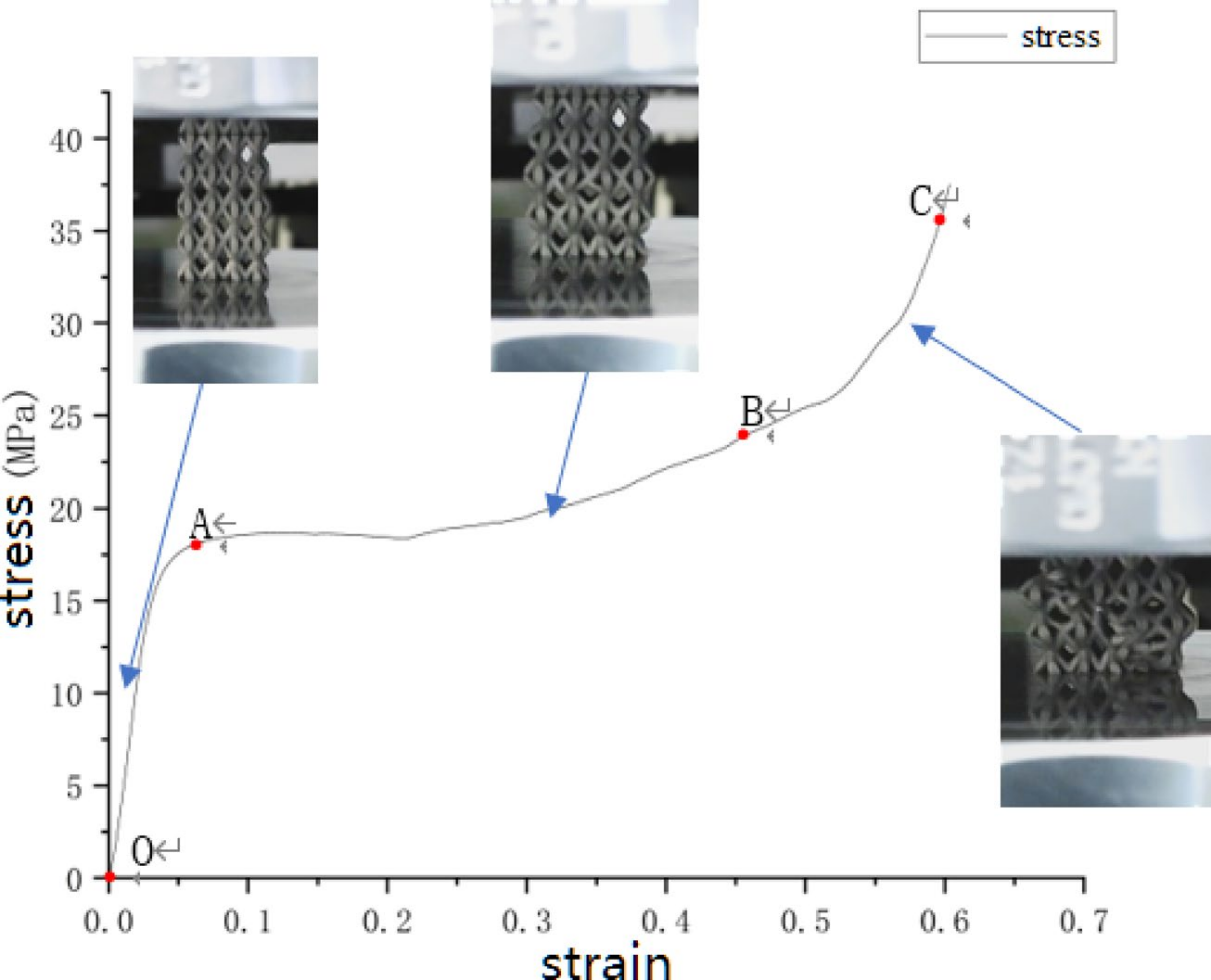
Compression process of lattice structures.

In this experiment, 17 groups of BCT lattice structures with different structural parameters were subjected to compression tests. These experimental designs aimed to explore the influence of different parameters on the compression performance of the material. During the experiments, due to the inherent high plasticity of 316L stainless steel, none of the specimens fractured throughout the entire compression process. In the initial stage of the experiment, the compression stress experienced by the specimens was low, resulting only in elastic deformation with the structure remaining relatively intact. As the compression stress, i.e., the load from the compression machine, gradually increased, visible plastic deformation began to appear in the rods of the middle upper and lower layers of the compressed specimens. The rods at the nodes gradually reduced their inclination angles relative to the compression plane as compression progressed. As the compression displacement increased, the material at the upper and lower nodes was compressed and entered the yield state. This resulted in a decreased load-bearing capacity and further reduction in the inclination angles of the lattice structure’s rods. When the applied compression reached a critical level, the deformation of the rods in each layer saturated, and the lattice structure became fully compacted. Observations from the load application and compression process images of the printed specimens reveal that the specimens did not fracture after compression, as shown in Fig. 3: Compression Process of Lattice Structures.

Analyzing the deformation characteristics of the compressed specimens, the process can be divided into three stages: elastic deformation stage, yield stage, and densification stage.

- *Elastic Deformation Stage (OA)* During this stage, the BCT lattice structure experiences low-level compression stress, exhibiting elastic behavior. This indicates a good linear relationship between stress and strain, consistent with Hooke’s Law.
- *Yield Stage (AB)* As the load continues to increase, the structure begins to undergo localized plastic deformation. In this stage, while strain continues to increase, the stress levels plateau.
- *Densification Stage (BC)* The BCT lattice structure becomes compacted under the current compression. The deformation of the specimen is highly significant, with overlapping support rods in each layer leading to a sharp increase in stress.

The experimental data obtained from the validation experiments were converted into stress-strain curves. These curves clearly illustrate the mechanical behavior of the material during compression, including the elastic stage, yield point, and subsequent deformation stages. By analyzing the stress-strain curves, the YS and MOE of the lattice structures under different structural parameters can be determined. In the validation experiments, structural parameters were altered based on the aforementioned groupings to study their impact on the mechanical performance of the lattice structures. The YS and MOE data of the lattice structures under different structural parameters are presented in Table 4.

**Table 4:** Response surface experimental scheme and experimental results.

No.	D (mm)	L (mm)	θ(°)	YS (MPa)	MOE (MPa)
1	1.5	4.0	60	169.99	2802.34
2	0.5	5.0	45	0.82	12.42
3	0.5	4.0	60	3.05	89.67
4	1.5	4.0	30	19.79	805.21
5	1.0	4.0	45	21.47	490.26
6	1.0	5.0	30	5.08	63.41
7	1.0	4.0	45	21.47	490.26
8	1.0	4.0	45	21.47	490.26
9	0.5	4.0	30	1.71	28.68
10	1.5	3.0	45	93.03	2373.11
11	1.0	3.0	30	12.87	708.75
12	1.0	4.0	45	21.47	490.26
13	0.5	3.0	45	5.10	64.74
14	1.5	5.0	45	34.52	644.38
15	1.0	5.0	60	16.78	349.73
16	1.0	4.0	45	21.47	490.26
17	1.0	3.0	30	12.87	708.75

It is worth noting that both the numerical simulations and experimental tests consistently identify the same structural parameter combination (D = 1.5 mm, L = 4 mm, and θ = 60°) as yielding the maximum yield strength and modulus of elasticity. This consistency demonstrates that the finite element model is capable of correctly capturing the dominant mechanical trends and identifying optimal structural configurations for BCT lattice structures.

Nevertheless, certain discrepancies between the predicted and measured values can be observed. These differences are mainly attributed to manufacturing-induced defects inherent to the selective laser melting process, which are not explicitly incorporated into the idealized finite element models. Surface roughness and geometric imperfections of struts may lead to premature local yielding and enhanced stress concentration, while internal porosity effectively reduces the stiffness and load-bearing capacity of the lattice. Similar defect-sensitive mechanical responses have been widely reported for additively manufactured lattice structures, where deviations between simulation and experimental results were closely associated with manufacturing quality and defect distribution.

Despite these discrepancies, the finite element simulations successfully reproduce the relative influence of key structural parameters on the mechanical performance of BCT lattice structures and accurately predict the locations of optimal parameter combinations. This indicates that the proposed modeling framework remains reliable for comparative analysis and structural optimization, even in the presence of unavoidable manufacturing imperfections.

### Analysis of the influence of different structural parameters on the YS of lattice structures

In this validation experiment, RSM was utilized to construct a predictive model that visually and quantitatively demonstrates the impact of different structural parameters on the YS of 316L stainless steel BCT lattice structures. The established model, presented in the form of interaction surface plots, provides a visual representation of the relationships between each structural parameter and YS. These surface plots illustrate that increases in unit cell rod diameter and rod inclination angle have a positive effect on yield strength, whereas an increase in rod length negatively impacts mechanical performance. By inputting the experimental data into the pre-designed response surface model, the performance under different structural parameters can be predicted. Utilizing the response surface model, interaction surface plots are generated to intuitively show the interactions between different factors affecting YS and MOE.

**Fig. 4:**
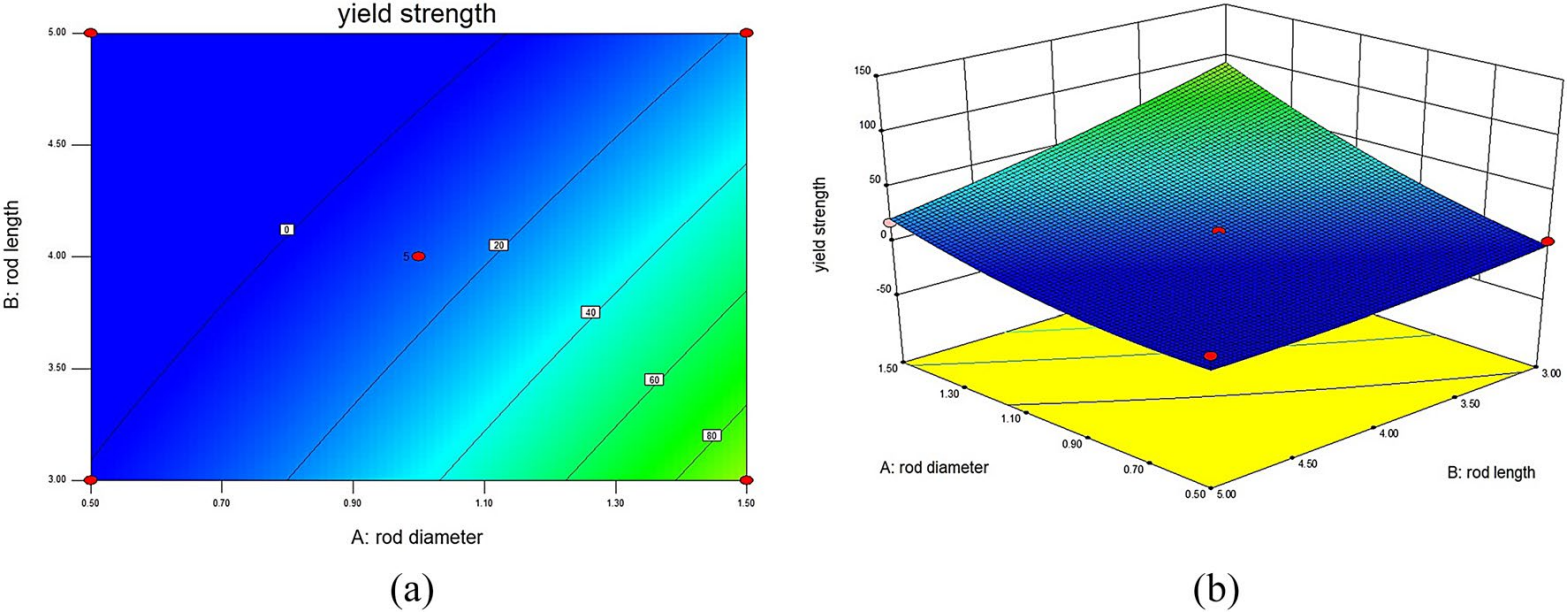
Interaction contour and response surface plots for YS DL: (**a**) contour plot of YS interaction between D and L; (**b**) response surface plot of YS interaction between D and L.

In Fig. 4, which illustrates the interaction contour and response surface for YS between rod diameter (D) and rod length (L) with θ set at 45°, it is evident that when L remains constant and D increases, YS gradually increases. Conversely, when D remains constant and L increases, YS decreases gradually. When D increases while L decreases, the surface exhibits a larger variation, indicating that the load-bearing capacity of the internal rods of the lattice structure increases, leading to a gradual increase in YS. Significantly, when D is 1.5 mm and L is 3 mm, the YS of the lattice structure reaches a maximum of 93.03 MPa. However, when L is 5 mm, the increase in D results in a smaller variation in the surface, indicating a relatively minor impact on mechanical performance.

**Fig. 5:**
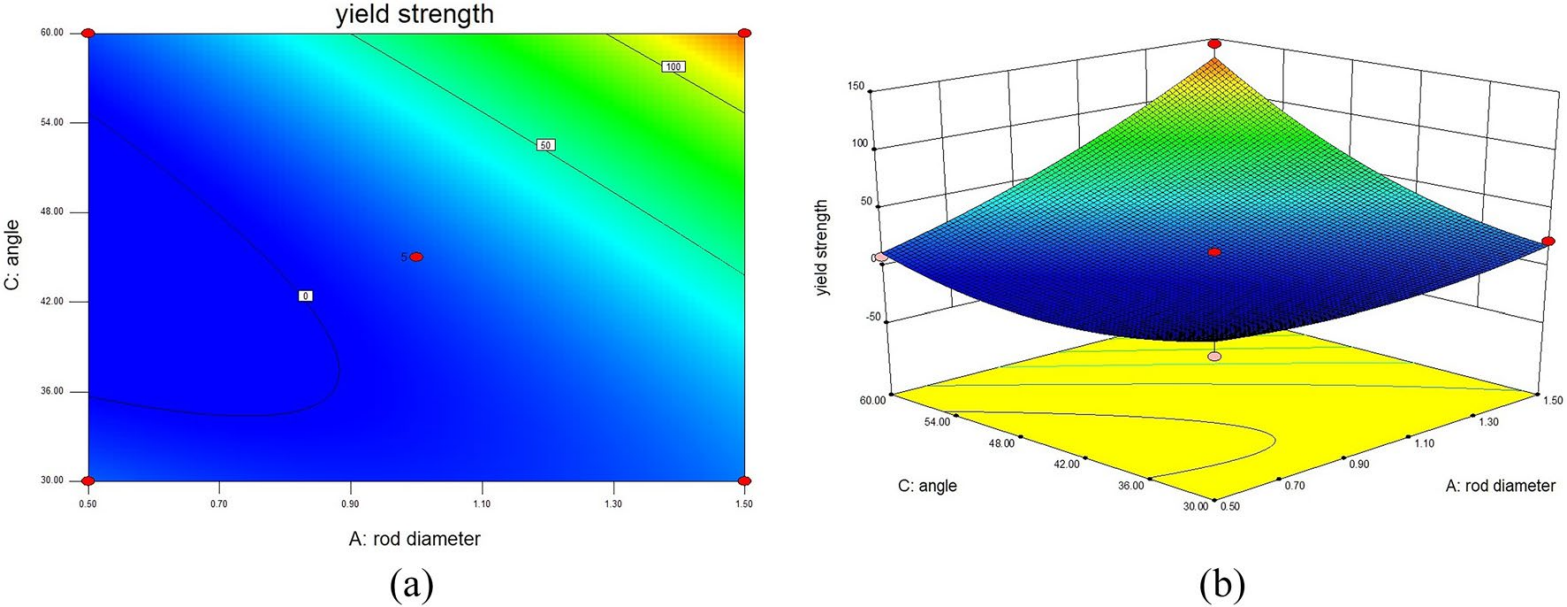
Interaction contour and response surface plots for YS of D and θ: (**a**) contour plot of YS interaction between D and θ; (**b**) response surface plot of YS interaction between D and θ.

In Fig. 5, which presents the interaction contour and response surface for YS between rod diameter (D) and angle (θ) with L set at 4 mm, it is observed that when either D or θ remains constant, the YS of the lattice structure increases with the increase of the other factor. When both D and θ increase simultaneously, the response surface shows a significant upward trend, particularly in the central internal structure of the lattice, enhancing the resistance to deformation and resulting in a substantial increase in YS. This indicates that D and θ have a significant impact on the mechanical performance of the lattice structure. Specifically, when D is 1.5 mm and θ is 60°, the YS of the lattice structure sample reaches a maximum of 169.99 MPa.

**Fig. 6:**
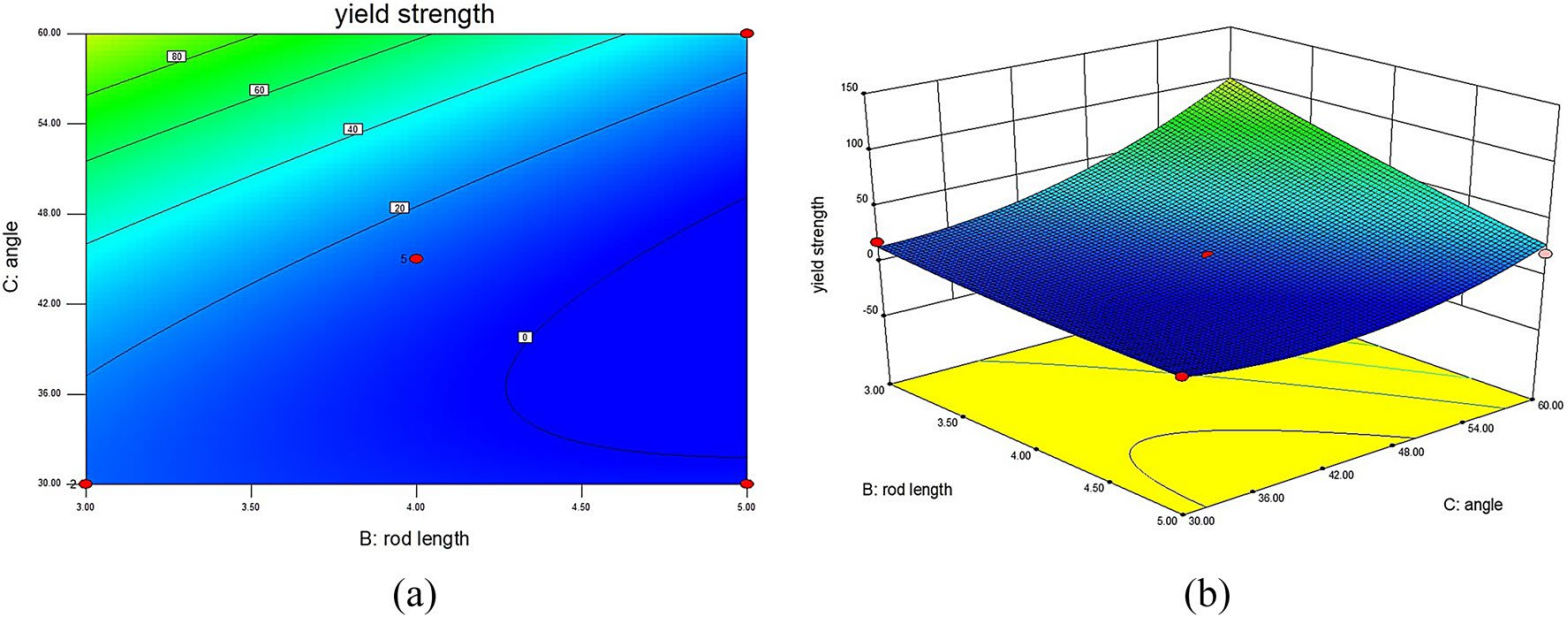
Interaction contour and response surface plots for YS L and θ: (**a**) contour plot of YS interaction between L and θ; (**b**) response surface plot of YS interaction between L and θ.

In Fig. 6, illustrating the interaction contour and response surface for YS between rod length (L) and angle (θ) with D set at 1.5 mm, it is evident that when L remains constant and θ increases, the load-bearing capacity of the internal rods of the lattice structure is enhanced, resulting in an increased YS. When θ increases while L decreases, the surface exhibits an upward trend, leading to an increased YS. Specifically, when L is 4 mm and θ is 60°, the YS of the lattice structure sample reaches a maximum of 169.99 MPa.

The curvature of the response surfaces indicates a strong interaction effect among the geometric parameters, suggesting that the mechanical performance of BCT lattice structures cannot be optimized by adjusting a single parameter independently.

### Influence of different structural parameters on the MOE of lattice structures

This experiment utilizes RSM to establish a model that visually demonstrates the impact of different structural parameters on the MOE of 316L stainless steel BCT lattice structures. The interaction surface plots generated through this method clearly show the interrelations between each structural parameter and the MOE. The different surface plots reveal that unit cell rod diameter and rod inclination angle have a positive influence on the MOE: as the unit cell rod diameter increases, the MOE of the structure also increases. Similarly, an increase in the rod inclination angle leads to an increase in the MOE. The surface plots also indicate that unit cell rod length has a negative impact on the MOE: as the unit cell rod length increases, the MOE gradually decreases.

**Fig. 7:**
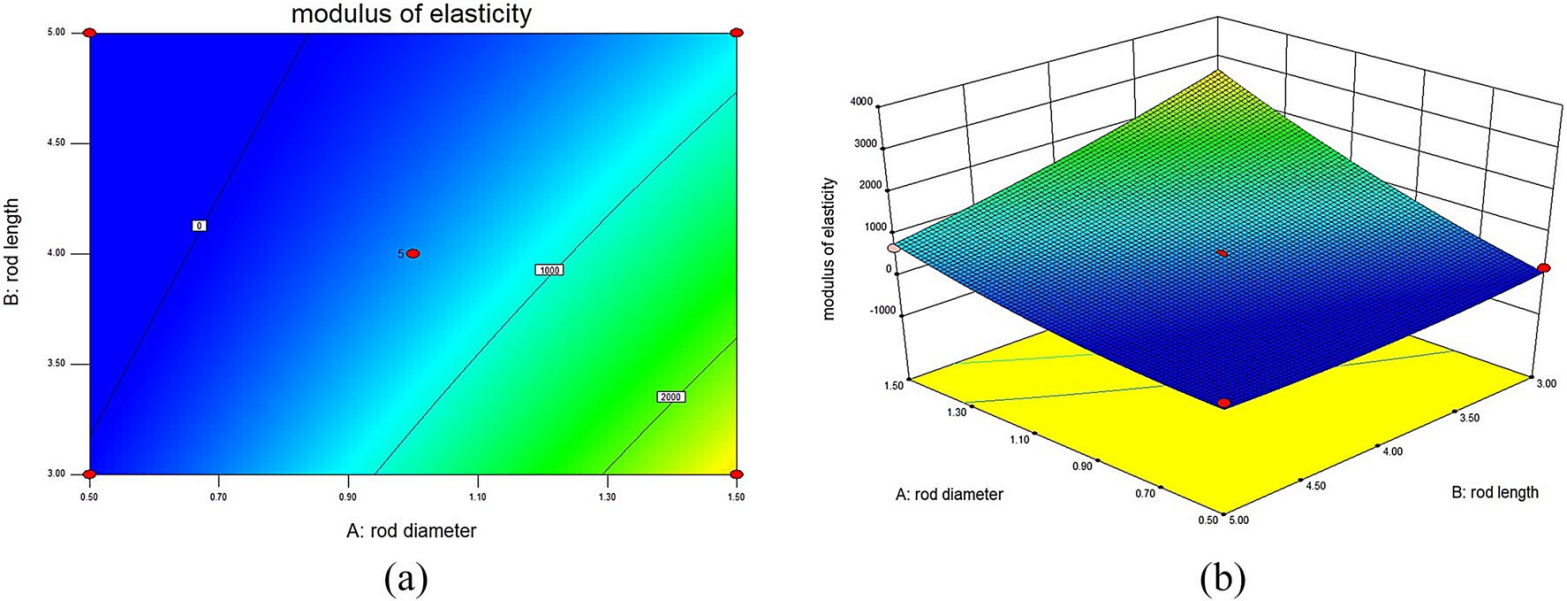
Interaction contour and response surface plots for MOE DL: (**a**) contour plot of MOE interaction between D and L; (**b**) response surface plot of MOE interaction between D and L.

In Fig. 7, depicting the interaction contour and response surface for MOE between rod diameter (D) and rod length (L) with θ set at 45°, it is clear that when L remains constant and D increases, the MOE gradually increases. Conversely, when D remains constant and L increases, the MOE decreases gradually. When D increases while L decreases, the surface exhibits a larger variation, indicating that the load-bearing capacity of the internal rods of the lattice structure increases, leading to a gradual increase in MOE. Significantly, when D is 1.5 mm and L is 3 mm, the MOE of the lattice structure reaches a maximum of 2373.11 MPa. However, when L is 5 mm, the increase in D results in a smaller variation in the surface, indicating a relatively minor impact on mechanical performance.

**Fig. 8:**
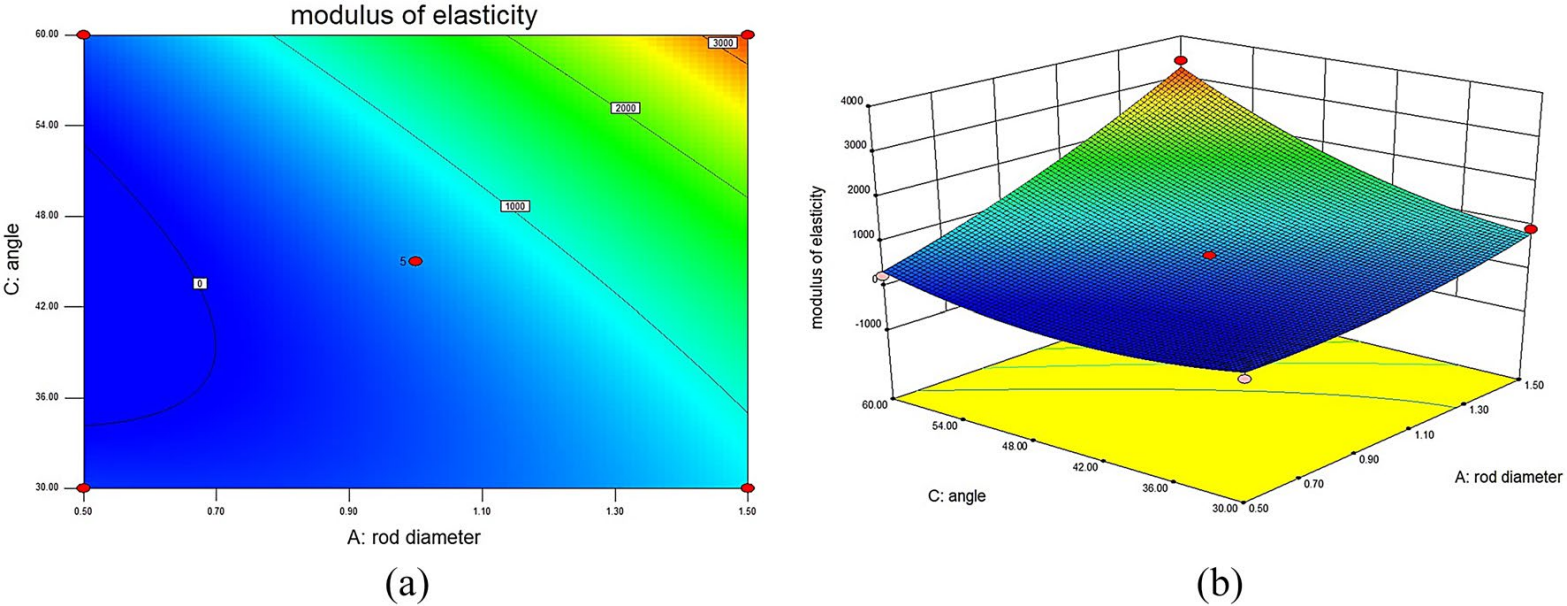
Interaction contour and response surface plots for MOE D_θ_: (**a**) contour plot of MOE interaction between D and θ; (**b**) response surface plot of MOE interaction between D and θ.

In Fig. 8, illustrating the interaction contour and response surface for MOE between rod diameter (D) and angle (θ) with L set at 4 mm, it is observed that when either D or θ remains constant, the MOE of the lattice structure increases with the increase of the other factor. When both D and θ increase simultaneously, the response surface shows a significant upward trend, particularly in the central internal structure of the lattice, enhancing the resistance to deformation and resulting in a substantial increase in MOE. This indicates that D and θ have a significant impact on the mechanical performance of the lattice structure. Specifically, when D is 1.5 mm and θ is 60°, the MOE of the lattice structure sample reaches a maximum of 2802.34 MPa.

**Fig. 9:**
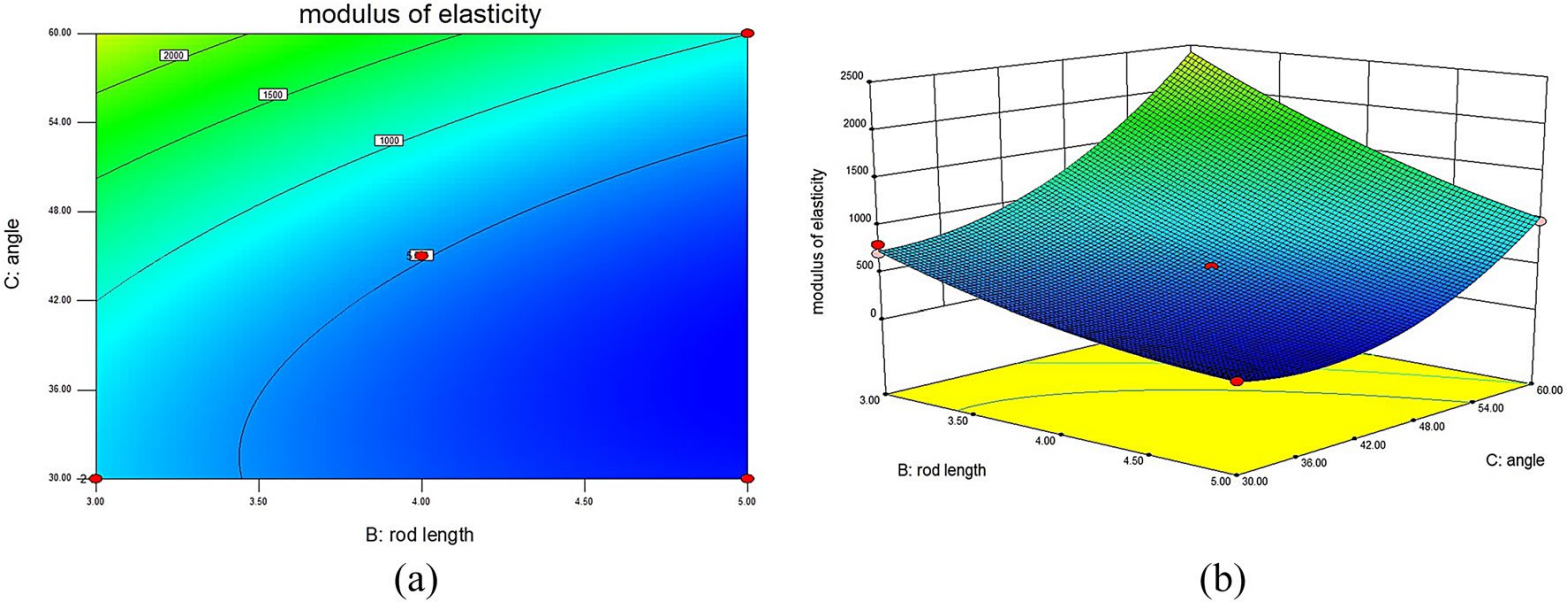
Interaction contour and response surface plots for MOE Lθ: (**a**) contour plot of MOE interaction between L and θ; (**b**) response surface plot of MOE interaction between L and θ.

In Fig. 9, depicting the interaction contour and response surface for MOE between rod length (L) and angle (θ) with D set at 1.5 mm, it is evident that when L remains constant and θ increases, the load-bearing capacity of the internal rods of the lattice structure is enhanced, resulting in an increased MOE. When θ increases while L decreases, the surface exhibits an upward trend, leading to an increased MOE. Specifically, when L is 4 mm and θ is 60°, the MOE of the lattice structure sample reaches a maximum of 2802.34 MPa.

These results further confirm the existence of significant interaction effects among structural parameters, highlighting the necessity of multi-parameter optimization strategies, such as response surface methodology, for the rational design of lattice structures.

## Conclusion

The mechanical behavior of 316L stainless steel BCT lattice structures fabricated by selective laser melting was investigated through numerical simulation, experimental validation, and response surface methodology. The main conclusions are summarized as follows:

1. The yield strength (YS) and modulus of elasticity (MOE) of BCT lattice structures are strongly governed by geometric parameters. Increasing rod diameter and rod inclination angle significantly enhances both YS and MOE, whereas increasing rod length leads to a gradual deterioration in mechanical performance.
2. Both simulation and experimental results consistently identify an optimal structural configuration (rod length = 4 mm, rod diameter = 1.5 mm, and inclination angle = 60°), at which the BCT lattice structures exhibit the best mechanical performance within the investigated design space.
3. Response surface methodology effectively captures the interaction effects among structural parameters and provides accurate quantitative predictions of YS and MOE, demonstrating its suitability as a design-oriented optimization tool for lattice structures.
4. The good agreement between numerical simulations and experimental results confirms the reliability of the proposed combined approach, and the obtained findings offer practical design guidance for lightweight and high-performance BCT lattice structures fabricated by additive manufacturing.

## Data Availability

The datasets generated and/or analysed during the current study are available from the corresponding author on reasonable request.
